# An Active Inference Account of Touch and Verbal Communication in Therapy

**DOI:** 10.3389/fpsyg.2022.828952

**Published:** 2022-05-20

**Authors:** Joohan Kim, Jorge E. Esteves, Francesco Cerritelli, Karl Friston

**Affiliations:** ^1^Department of Communication, Yonsei University, Seoul, South Korea; ^2^Clinical-Based Human Research Department, Foundation Center for Osteopathic Medicine Collaboration (COME) Collaboration, Pescara, Italy; ^3^Malta ICOM Educational Ltd., St. Julian’s, Gzira, Malta; ^4^Research Department, University College of Osteopathy, London, United Kingdom; ^5^Wellcome Centre for Human Neuroimaging, Institute of Neurology, London, United Kingdom

**Keywords:** active inference, abduction, guided touch, Markov blanket, communication, interoception, meditation

## Abstract

This paper offers theoretical explanations for why “guided touch” or manual touch with verbal communication can be an effective way of treating the body (e.g., chronic pain) and the mind (e.g., emotional disorders). The active inference theory suggests that chronic pain and emotional disorders can be attributed to distorted and exaggerated patterns of interoceptive and proprioceptive inference. We propose that the nature of active inference is abductive. As such, to rectify aberrant active inference processes, we should change the “Rule” of abduction, or the “prior beliefs” entailed by a patient’s generative model. This means pre-existing generative models should be replaced with new models. To facilitate such replacement—or updating—the present treatment proposes that we should weaken prior beliefs, especially the one at the top level of hierarchical generative models, thereby altering the sense of agency, and redeploying attention. Then, a new prior belief can be installed through inner communication along with manual touch. The present paper proposes several hypotheses for possible experimental studies. If touch with verbal guidance is proven to be effective, this would demonstrate the relevance of active inference and the implicit prediction model at a behavioral level. Furthermore, it would open new possibilities of employing inner communication interventions, including self-talk training, for a wide range of psychological and physical therapies.

## Introduction

One of the main arguments of the present paper is that the essence of therapy is an intervention to alter patients’ habituated interpretation of their interoceptive signals, which may underwrite chronic pain and emotional disorders. Manual touch can be a potent and effective tool in this process, because it can provide new interoceptive signals and promote a therapeutic (re)interpretation (guidance) of interoceptive signals (touch).

The main purpose of this paper is to propose the following hypotheses: a new and therapeutic framework of (re)interpretation can be instantiated efficiently through verbal communication. To facilitate therapeutic (re)interpretation, we argue for methods that enable the redeployment of attention, flattening prior beliefs, thereby weakening pre-existing generative models, and installing new generative models. It should be noted that the aims of this paper are purely hypothetical: this paper is not a comprehensive review of what is currently known about guided touch, rather, it introduces some novel hypotheses, based on the active inference framework. The ensuing hypothetical framework explains why guided touch could undergird effective therapy for the treatment of chronic pain and emotional disorders.

The enactivistic perspective proposes that pain and emotion can be attributed to active inference, or the top-down and bottom-up communication processes among levels of neuronal hierarchies. The human body generates enormous amounts of interoceptive signals from myriads of organs including guts and heart; most of the signals are ignored and processed at unconscious levels, but some “unusual”—that is, unpredicted or unattenuated—signals receive special attention, revising beliefs at higher levels of processing that may include conscious processing. The “unusual” signals drawing stronger attention are often interpreted as bad feelings, negative emotions, or pain.

Tons of interoceptive sensory data are continuously arriving, but most are effectively ignored in the sense that they convey no useful information—or they are returned to the active states immediately to engage various reflexes, without any further (central) processing. One of the important tasks for the brain’s active inference system is to filter out, attenuate and ignore the uninformative sensations.

Enactivism suggests that the goal of manual therapy should be about improving active inference through re-construction of the sense and meaning making from sensations. On this view, through touch, the manual therapist should equip the patient with new ways of interpreting interoceptive and proprioceptive stimuli. From this perspective, the present paper proposes, in order to help patients with persistent physical symptoms or emotional disorders, therapists should “install” new prior beliefs for alternative ways of interpreting bodily sensations and new ways of sense-making. The “installation” may take various forms, from strong “implantation” to gentle “suggestions,” all depending on the context in which pain and emotion are experienced by patients (Von Mohr and Fotopoulou, 2018).

Although the present paper is not strictly enactivist, the proposed active inference framework is, in fact, aligned with the enactivist approach to cognition (Parr et al., 2022). Moreover, we also have proposed osteopathic care (a form of hands-on care) as (En)Active inference (Esteves et al., 2022). In this vein, the present paper proposes that touch therapy is a dynamic interactive ritual that provides opportunities for reinterpreting sensory signals, redeploying attention, and attenuating and ignoring irrelevant or ego-dystonic sensory inputs.

The enactivistic and predictive model of active inference maintains that emotion and pain are inferences or explanations for “states of being” that are based on largely interoceptive signals. Theories in social neuroscience suggest that interoceptive sensations may arise from other people as well as from the body (Cacioppo et al., 1992; Norman et al., 2014). The somatovisceral afference model of emotion (SAME) demonstrates that touch may produce “social interoception.” The implication is that touch—with certain characteristics carried by thin, unmyelinated fibers called C-tactile afferents (CTs) to the posterior insula—supplies interoceptive signals that may have strong influences on emotional experience (Burleson and Quigley, 2021).

Active inference explains sentient behavior in terms of inference under a generative model that can generate the sensory consequences of some hidden or latent states of being. Crucially, the enactive aspect of active inference rests on closing the action perception cycle by considering the role of action in generating sensations. This action can be overt—e.g., moving muscles or engaging autonomic reflexes—or covert—e.g., redirecting attention so that messages from sensory organs are augmented or attenuated. The implicit circular causality between the internal states of the brain and the external states of the body (and environment) is based on the notion of a Markov blanket.

Following Ramstead et al. (2019), we consider the human body as a recursively nested collection of Markov blankets. Our Markov blanket comprises sensory and active states (e.g., sensory epithelia and the motor system, respectively). Its role is to mediate exchange between internal states (e.g., neuronal activity) and external states (e.g., physiology within the body and states of affairs within the world). Sensory states mediate the effect of external states on internal states (i.e., perception) while active states mediate the effect of internal states on external states (i.e., action). In dyadic (e.g., practitioner patient) interactions, the sensory states of one Markov blanket become the active states of the other and *vice versa*.

The ultimate goal of touch therapy in general is to help patients develop new internal models for interpreting the interoceptive signals (Von Mohr and Fotopoulou, 2018; Cerritelli et al., 2020). Touch therapy is a process of establishing shared narratives through “listening to body-talk and constructing body-stories” (Gale, 2011). In this therapeutic process, the therapist suggests new possibilities—or hypotheses—to explain patterns of sensory input; in other words, encouraging the patient to experience (i.e., explain) somatic sensations in a way that eludes entrenched explanations, such as “I am suffering.”

Touch therapies, and for that matter any other types of therapies as well, should help patients to establish a revised version of their self model; that is, new prior beliefs, new hypotheses, and new generative models. Persistent physical syndromes such as chronic pain, and emotional disorders (anxiety, depression, trauma stress, etc.) can be attributed to aberrant active inference, in which certain hypotheses (e.g., “these visceral sensations are evidence that I suffer from chronic pain”) become self maintaining. To revise their self-maintaining hypotheses, patients need to discard old and erroneous habits of bad interpretation (i.e., false inference) and establish new and healthy ways of interpreting their interoceptive signals. In this setting, manual therapies can be a very effective treatment, because touch can directly provide new sensory signals in a context that calls for a novel (i.e., therapeutic) interpretation.

From the enactivistic perspective, the present treatment attempts to answer the following questions: how can we effectively replace the old and disabling generative model of a patient with a new and enabling model through touch therapy? Why is guided touch (touch accompanied with verbal communication) necessary? What types of guidance would enhance the effects of the manual therapy and strengthen the therapeutic alliance? And why? To test the validity of putative answers, we propose a set of hypotheses for possible empirical studies.

The authors of the present paper envisage that the strategic methods of manual touch with verbal guidance would benefit the providers of mental and physical health care services through hands-on techniques, such as osteopaths (Bohlen et al., 2021; Esteves et al., 2022; McParlin et al., 2022), somatic psychotherapy (Guest and Parker, 2022), body-centered psychotherapy, Hakomi Method, sensorimotor psychotherapy (Fisher, 2019), rolfing (Micozzi, 2018), Functional Integration in Feldenkrais Method (Bearman and Shafarman, 1999), hands-on in Alexander techniques (Carneiro et al., 2020), and Somatic Experiencing (SE) therapy (Payne et al., 2015).

## Active Inference and Markov Blankets

### Considering the Body as a Markov Blanket

Life is the continuous exchange of energy and material between the inside and outside, without fully isolating the inside from the outside (environment). Without boundaries, there is no life. Indeed, one could argue without boundaries there would be no-thing because everything would be the same thing. The “boundary” itself has probabilistic, stochastic properties that change with the progress of time (Friston, 2012). This boundary can be described technically as a Markov blanket. The very existence of a Markov blanket means that the internal states effectively minimize the free energy (i.e., implausibility and dispersion) of their blanket states. In other words, active and internal states will appear to maintain homeostasis and autopoiesis through Bayesian reasoning (Friston, 2013).

Statistically speaking, the Markov blanket is about a network of multiple states, influencing one another, statistically linked together with Markov chains. In discussing graphical models that depict probabilistic relationships between states and events (nodes), Pearl (1988) defines a Markov blanket as follows: the minimum set of nodes required to sufficiently predict the state of a specific node is the Markov blanket of that node. It looks like a “blanket” as it surrounds and statistically insulates the node in question. This statistical insulation means that all the information necessary to predict the next state of the node is contained in its blanket nodes or states.

**FIGURE 1:**
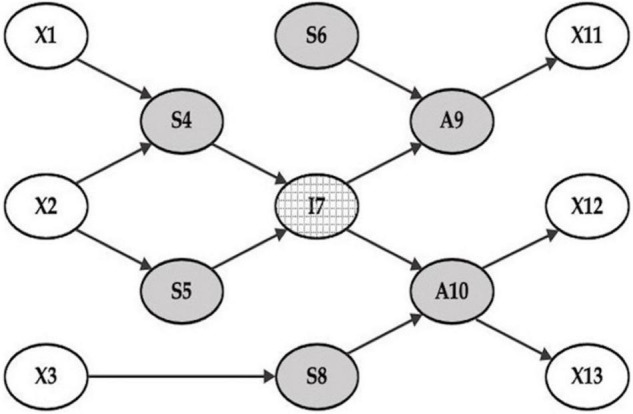
A Markov blanket. A Markov blanket of a particular node probabilistically defines the state of a particular node without being affected by the state of any other nodes. The external states are outside the Markov blanket (the nodes of X1, X2, X3, X11, X12, X13), which are the environment or the world (and body) in which we live. The internal state, or I7, is what is inside—the neuronal and possibly conscious self. The Markov blanket consists of the gray circles surrounding the internal state I7. Among them, the nodes A9 and A10 are active states influenced by the internal state and influencing the environment, or the external states (X11, X12, and X13). The rest are the sensory states, and there are two types: one that provides the sensory data to the internal state (S4 and S5), such as sensory organs producing perceptions; the other directly influences the active states without going through the internal state (S6 and S8), such as automated sensory-motor reflexes processing information at a sub-personal level, unconsciously. The human body, comprising sensory states (S4, S5, S6, and S7) and active states (A9 and A10), lies between the internal state (i.e., sentience) and the external states (i.e., environment). Note that the arrows in this graphical model represent Markovian dependencies, referring to probabilistic effects, rather than strictly deterministic influences.

In Figure 1, “I7” is the specific (internal) node surrounded by S4, S5, S6, S8, A9, and A10. If the information about the boundary nodes is given, the state of I7 can be predicted (Kirchhoff et al., 2018). Any additional information about outside the blanket, such as node X1 or X13, does not contribute to the prediction of node I7.

Friston proposes that the Markov blanket construct entails the four states of any particle or organism: namely, the internal, external, sensory, and active states (Friston, 2010). It should be noted that “external states” here do not refer to external objects or the environments *per se*. Rather, it means states that are hidden from internal (neuronal) states by the blanket (sensory) states. In the context of interoception, these external states correspond to physiological and homeostatic states of the body. In other words, the “external states” are bodily states that have to be inferred.

By selectively sampling salient information from the sensory states, the internal state constructs and image of the outside world through a free energy or prediction-error minimizing processes (Friston, 2013). It is “active” inference because the sensory states depend upon active states. In other words, the very action of the active states (A9 and A10) on the external objects (say, X1, and X2), strongly influences the internal state’s interpretation of the sensory data (S4 and S5). The active state of the Markov blanket can change the environment and reduce the free energy of the Markov blanket itself by actively ensuring the sensory states are consistent with the predictions afforded by internal states. In short, active states maintain the structural-functional integrity of the Markov blanket. This can be read as an elemental form of autopoiesis in the sense that we organize and create ourselves (Varela et al., 2016).

When we make active inferences and minimize prediction errors (i.e., free energy), we do not necessarily call on conscious or declarative processes, e.g., “Oh, my prediction was wrong, I will correct it.” Most of the prediction error minimization processes are sub-personal, right down to the level of motor and autonomic reflexes that control movement and mediate physiological homeostasis, respectively. The sensory system is generally considered to be at the lowest level of a hierarchy of internal states that comprises several levels. Internal states such as neuronal activity and synaptic efficacy are driven by bottom-up or ascending prediction errors. These prediction errors can be read as free energy and are computed by comparing representations at each hierarchical level with top-down or descending predictions based on representations at higher levels. In other words, the internal states are hierarchically organized and influence each other through a counter stream of ascending prediction errors and descending predictions. The minimization of prediction error is automatically made at all levels of processing. The basic task of the brain is therefore to generate perceptual hypotheses and narratives about the world by constantly inferring the meaning of ambiguous and sparse sensory data. Technically, this prediction error minimization (a.k.a. predictive coding) is a particular instance of Bayesian belief updating under a hierarchical generative model, where deep hierarchical models entail deeply structured prior beliefs about how sensations are generated. One might imagine that at the deepest levels of hierarchical inference, hypotheses about selfhood, intentions and narratives—based on perceptions—could underwrite conscious processing.

In guided touch, the sensational information conveyed by touch would be mostly processed unconsciously at a sub-personal level, while accompanying attribution of agency and inferring the intentional or propositional stance of the therapist—in conjunction with verbal communication—would be the remit of higher-level processing. For example, the touch itself would work on S4 and S5, while the verbal guidance could directly influence I7.

### Scale Invariance and the Nested Structure of Markov Blankets

Markov blankets exist not only at the brain level: for a cell to survive as a living system, it needs a cell membrane as a boundary that separates itself from the surrounding milieu. Each cell is a Markov blanket. Each organ, such as the heart, stomach, and kidneys, also has boundaries, which are Markov blankets. For example, as an organ, the heart constantly exchanges information with other parts of the body, including the brain, while maintaining its own independent functions. The same goes for other organs, such as the kidneys and stomach. Each organ has a boundary that separates its internal states from its external states.

The human body, which is a collection of various kinds of organs, is also a Markov blanket. In addition, an organization or society formed by a group of humans can also be said to be a large Markov blanket. All countries maintain a boundary that separates the inside from the outside. So, a nation is also defined by a Markov blanket. In this way, one Markov blanket can act like a node in a larger Markov blanket, and at the same time, it could have a network of smaller Markov blankets within itself. This property is called the “nested structure” of the Markov blanket (Kirchhoff and Kiverstein, 2021), or “a multiscale nesting of Markov blankets that integrates the smallest scale of the cell to the largest scale of the embodied person” (Kiverstein et al., 2022).

**FIGURE 2:**
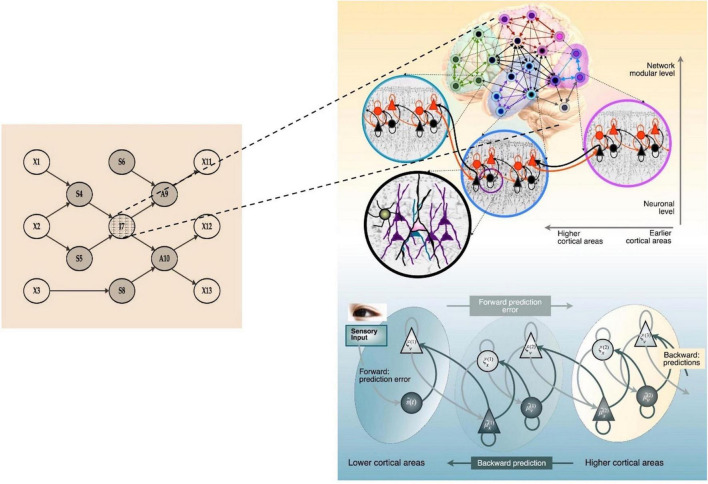
A Markov blanket in the multiscale hierarchical organization of brain networks. The internal state, I7, of a Markov blanket has a hierarchical structure for minimizing prediction errors. In the right panel, red and bright triangles represent prediction errors from bottom-up sensory signals and black and dark triangles indicate top-down predictions (updated by prediction errors) under the implicit (deep or hierarchical) generative model. The process of hierarchical prediction, or deep active inference, may exist at various scales: between brain regions, between smaller nodes within a brain region, and even at the neuronal level. The Markov blanket is basically scale invariant; the nodes in the model could be cells, neurons, cortical columns, brain areas, organs in the body, individuals, organizations, or nations. The right panel image was adopted from Park and Friston (2013).

Not only is the structure of the Markov blanket nested, but the functional networks of active inference can be also nested. In other words, the nested structure of the Markov blanket can be found in functional networks as well as in structural connections. As such, the arrows in the Markov blanket model represent functional as well as structural connectivity. Park and Friston (2013) have emphasized the similarity between hierarchical topologies (based on structural and functional connectivity among the brain regions) and the effective connectivity that would be required for hierarchical message passing (at the neuronal level) required for active inference. The pursuing hierarchical and the nested structures are illustrated in Figure 2.

### Understanding Consciousness Through the Markov Blanket

At the highest level of hierarchically organized internal states, conscious processing may correspond to an image of the self that integrates inferences about the world with its own action (Friston et al., 2010). The information about action *per se* is supplied by proprioceptive and interoceptive sensations. When the action is related to joints, fascia, and muscles, it produces proprioception; when the action, usually unconscious, is related to internal organs such as the heart and guts, it produces interoception. Conscious (and unconscious) inference therefore entails continuous sampling and resampling of the sensory data, sometimes ignoring and other times augmenting what it samples, to produce plausible inferences about the world and predictive regulations.

Consider the relationship between X1 and S4 in Figure 1. X1 is an external object in the environment. S4 is a sensory organ, which influences internal states to produce statistically plausible percepts regarding X1. The structure of the relationship between the internal representation of X1 and S4 is multi-layered and hierarchical; there are continuous interactions between the top-down predictions of the internal model and the bottom-up prediction errors that drive belief updating (for example in the right-hand panel of Figure 2 that unpacks the internal states in I7). Through these interactions, X1 (external objects) becomes X1′ (percepts). The single set of states in I7 are, in fact, a collection of internal states with multi-layered hierarchical deep structures, which dynamically pass signals up and down, as shown in Figure 2.

Active inference under these deep models attempts to make meaningful stories from perceptions—stories or narratives that necessarily entail some action. For example, inferences about X1′, automatically inform possible actions to be made on X1 through the active states of A9 or A10. As such, any action affordances of X1 inevitably affect the active inference about X1′ reported by S4.

Assume that I see an apple (X1) on a table, and my visual system (S4) induces belief updating in my hierarchical generative model (X1′). In this situation, the various possibilities of my actions through A9 (biting, picking it up and smelling, or throwing it away) inevitably affect the process of active inference based on S4 (an apple as a percept) and deep within I7 (a delicious apple with full of meanings and memories, or affordances). This means that the possibility of my actions on a specific object affects my perception of that object. In turn, this is the meaning of “enactive cognition” or “cognition as embodied action” that Varela emphasized (Varela et al., 2016).

The active inference process starting from X1 → S4 → I7 → A9 → X1 evinces the self-referential loop of consciousness. It also shows that there are two basic functions of consciousness: one is attention (regarding S4) and the other is intention (regarding A9). It is notable that much of the information processed under the Markov blanket, however, may be processed at lower levels of unconscious inference.

As for consciousness, it is necessary to judge how accurately the percept (X1′) produced by S4 and the story (X1″) produced by I7 represent the real X1 “out there”; so that it can continuously update its internal model. The “accuracy” of the perception is to be judged not by how much the perception coincides with the actual and objective reality out there, but by how much such perception contributes to survival and reproduction of the perceiver—by minimizing prediction errors in all sensory modalities; including interoceptive domains that underwrite homeostasis and physiological viability. Theoretically speaking, there are at least four possible ways that consciousness can call on to test validity of its inferences and interpretations.

First is to continuously resample the sensory data from S4. For example, looking at the object again and again, perhaps from different perspectives, to confirm whether it is really an apple.

Second is to obtain other types or modalities of confirmatory sensory data. That is, comparing the visual data reported from S4 with the tactile or the olfactory data obtained by the other sensory organs, say, S5.

Third is to get additional information through A9 that acts on the apple. For example, picking it up and biting it, which would provide other types of sensory data updating the previous information.

Fourth is to cross-check whether the story (“This is an apple”) produced by consciousness is true. To check the validity of “my” story, consciousness needs stories produced by “other” consciousness—that means a Markov blanket always needs “other” blankets to complete the inference processes: As a storyteller, consciousness always presupposes others to communicate with. Thus, communicability underwrites the nature of consciousness.

This is why we always use language, a social convention, even when we think to ourselves. If “thinking to myself” is truly my own mental process, I would not need to rely on any language, a tool invented for communicating with other people. The fact that we have to rely on languages when we think to ourselves implies that “thinking to oneself” is not a purely isolated, personal, private mental process; rather it is inner communication of which fundamental nature is social.

Inner communication is what consciousness does. Consciousness always makes stories about one’s own experiences with languages that can readily be reported to others. Dehaene—the neuroscientist unearthing key issues of consciousness—also maintains that “consciousness” is to be distinguished from vigilance or attention, and the essence of consciousness is “conscious access.” In other words, consciousness is the agency that interprets and synthesizes the various experiences to produce something “reportable to others” (Dehaene, 2014, p. 8–9)—and therefore, reportable to me.

Consciousness is the process of constantly transforming my experiences into stories I may tell others (and myself). To validate my own story, “Oh, here is an apple,” I need someone to ask the question: “I see an apple. How about you? What do you see?” Consciousness, as a process of storytelling—of narrative sharing—presupposes the existence of others. Vis-à-vis other people, consciousness transforms itself into self-consciousness, which is a sense of agency (SoA) that produces my own stories and sense-making.

If, among the four methods listed above, the first three methods detect serious prediction errors, we could call them hallucinations. If the fourth method finds serious errors, we call them delusions. It is impossible for consciousness to judge on its own whether the perception it has inferred—or the story it has produced—is a veridical representation of reality. Consciousness becomes hallucinatory or delusional if it has a perceptual or narrative aspect that is significantly different from that of others. The criteria for evaluating the validity of conscious inference are not mathematics or logic. It can only be tested through communication with others. It is to be determined only by how far one is away from the reports of others’ inferences. If everyone is hallucinating or delusional, then no one is hallucinating or delusional. If I were the only creature on this planet, the concepts of hallucination or delusion would be irrelevant—indeed, I would not even need the notion of “self,” as distinct from “other.” This means that a Markov blanket needs other Markov blankets to communicate with, to engender selfhood and accompanying agency. We need another person. We need an alliance. In a therapeutic situation, we need it all the more.

From the standpoint of conscious inference (I7), important predictions must be made about the source of sensory information. Was it me or you who caused this sensation? I feel my arm is moving: Am I moving my arm or are you pulling it? I feel my foot is being lifted: Is my foot moving now as a result of my own leg movement, or is the ground that I am standing on starting to rise?

Determining the source of my sensations is a crucial issue in planning my further actions and predicting the consequences. When I walk with my arms and legs moving, the sensations from inside and outside are inherently mixed. If the source of movement or sensory generation is within me, I can plausibly infer that the movement is caused by an agent, or the “self.” This is why the self-consciousness of “I” is the inevitable result of the active inference “I am acting.” Intention to move and attention to movement are the fundamental sources of the SoA, or self-consciousness (Friston, 2017b). Intention and attention make up the self, not the other way around (Friston, 2018). We will see that guided touch is all about interpreting the intention of the therapist and directing the attention of the patient.

## Pain and Emotional Disorders – the Problem of False Inference

### On Pain

As a living organism, the human body cannot avoid constantly falling into surprising states, which cause certain (uncomfortable) interoceptive sensations. This “unpleasant feeling” is beneficial for survival as it urges active inference to respond to surprising situations and to restore balance by acting to sample familiar (comfortable) sensations. Some of the unpleasant feelings are interpreted as negative emotions (anxiety, panic, depression, and anger), others as pain. It is important, especially for therapists, to note that pain and emotions are the results of inference about interoceptive sensory data. In many cases, chronic pain occurs without any particular physiological reasons, which are often called functional or medically unexplained symptoms (MUS) (Calsius et al., 2016).

According to Friston (2017a), the sensory organs and the nervous system maintain a sort of gain control that emphasizes the important information while ignoring imprecise signals (e.g., sensory noise). However, when the gain control system is not working properly, the gain or “volume” of the miscellaneous sensory signals gets exaggerated and gains access to higher levels of processing and implicit inference. A lot of interoceptive sensory data that should have been ignored suddenly turn into salient and urgent signals, to be interpreted as signs of change that requires consciousness explanation. As a result, the patient experience things (i.e., perceives or infers) pain and emotional disturbances as the best explanation for interoceptive prediction errors that would normally be attenuated. In short, a failure to predict the precision of interoceptive prediction errors means it would be impossible to ignore certain sensations. And anything that cannot be ignored has to be explained.

From the perspective of active inference—and the accompanying precision control (Friston, 2017a)—we can read persistent functional symptoms and emotional disorders as a failure to neuromodulate the gain of sensory prediction errors. In other words, an inability to gate, attenuate or filter sensations in the usual way. Sensory attenuation is a vital part of action; perhaps the best example in saccadic suppression; namely, the attenuation of visual information during saccadic eye movements. Most chronic pain is a result of an inability to attenuate interoceptive prediction errors and attentional hypersensitivity to imprecise (i.e., noisy) nociceptive signals. Persistent functional symptoms may be caused by a mismatch between the prediction of pain [= p(pain | sensation)] and the likelihood of pain [= p(sensation | pain)]. Patients tend to interpret harmless or irrelevant sensations as evidence for pain. It is a failure to shift attention from the sensation that underlies the experience of pain (Hechler et al., 2016). In other words, a failure of disattention, rather than a failure of attention—a loss of ability to attenuate, and as such, a loss of capacity to render one’s body comfortably invisible. Thus, bodily symptoms should be considered in the context of “action and attention selection dynamics” (Pezzulo et al., 2019).

### On Emotion

The same is true with emotional disorders. Patients with emotional regulation disorders, such as anxiety or depression tend to interpret meaningless sensations as the “somatic markers” of the negative emotions (Damasio, 1999). For a healthy normal person, such prediction errors would be immediately corrected through cross-checking with other sensory information. However, the Markov blanket of patients would not be able to attenuate prediction errors optimally; rather, it augments all kinds of sensory prediction errors and gets caught up in a vortex of increasing anxiety that it is a situation of great uncertainty, anger, or angst.

There may be various physiological causes of this particular malfunction of inference, such as metabolic or immunologic issues, or hormonal imbalances and neurotransmitter imbalances. Of course, past trauma and traumatic memories can also cause false inference; particularly if the precision of prior beliefs (established during a traumatic experience) cannot be attenuated or revised. On this view, chronic pain and emotional regulation disorder share a common cause—a failure to attenuate precision (Limanowski and Friston, 2020). Therefore, the basic approaches for treating pain and emotion should share one feature: discarding the old and bad inference patterns and introducing new and good explanations for the interoceptive signals. Crucially, this will rest on re-establishing attentional control and selecting the right sensory data, on which to base new hypotheses about the embodied self.

Traditionally, psychology has viewed emotion as a preparatory stage for specific goal-directed behaviors (Moors and Fischer, 2019). But modern neuroscientists and psychologists regard emotion itself as a specific type of action: for example, Llinás (2002) considers emotion as a fixed action pattern, Seth and Friston (2016) consider emotion as derived from active inference based on interoceptive data, and Barrett (2017) as an integrated adaptive behavior of the body for allostasis.

According to Barrett’s integrative and constructivist approach, which is largely based on the prediction model and enactivism, emotions arise from interoceptive signals occurring during allostasis processes that regulate the body’s metabolism and energy. In other words, emotions are natural results of surviving in the environment as a human being with a body (Barrett et al., 2016).

From the enactivism perspective, Barrett strongly criticizes the notion of distinctive and individual emotions, such as fear and anger, each having their own entity. She maintains that emotions arise in the process of the body’s overall active inference and the nature of all negative emotions are formally identical. The distinctive negative emotions have merely been socio-culturally assigned and constructed (Barrett, 2017). Thus, it is likely that any treatments aimed at controlling an “individual” emotion will prove ineffective. Rather, it will be of great help to attenuate prediction errors that drive inference processes in the interoceptive and proprioceptive domains.

### Redeployment of Attention

Traditionally, pain has been regarded as a sign of some physiological problems. Therefore, if the broken parts of the body are fixed, with drugs or other treatments, the pain should go away. For some cases, especially with acute pain, this mechanistic understanding of pain would work. But there are many cases that the traditional mechanistic view cannot explain: for example, pain can be felt without any bodily dysfunctions; pain may disappear with placebo or even with sham treatments.

Active inference, however, can readily explain these phenomena. Pain occurs when our brain interprets the body as being in pain (Ongaro and Kaptchuk, 2019). Pain is created by the interaction between the internal (generative) model and incoming sensations. The dynamic relationship between the painful experience (and reported symptoms) and the objective status of the body are always different, depending on the individual, context, and culture (Van den Bergh et al., 2017).

If emotional disorders and chronic pain are caused by aberrant active inference processes, the treatment should focus on correcting the old and bad habits of inaccurate inference, by guiding the patient’s inference, not to pay too much attention to imprecise sensory data. This can be done by drawing attention from unattenuated sensations. This is what Friston (2017a) calls “redeployment of attention,” or recovering the ability of ignoring not-so-important sensory data. In this way, the inference system, or generative model, may attenuate and silence the volume of unnecessary sensory data, which could be mistakenly interpreted as pain or negative emotions.

It should be noted that the concept of (redeployment of) attention is distinct from the notion of distraction at a subjective or cognitive level. The term attention – in the active inference paradigm – refers to (usually sub-personal) encoding of the precision (i.e., confidence) of random variables, rather than to any cognitive attention at the conscious level (c.f., the distinction between exogenous and endogenous attention). As such, “paying attention to the sensory data” does not necessarily mean any intended and conscious attention. It is rather sub-personal and unconscious automated attentional selection at the lower (e.g., sensory) levels of the neuronal hierarchy. In short, the (re)deployment of attention is not something we consciously intend to do (as with saccadic suppression), but it can be learned (and possibly mentalised) through training.

A good example of such attentional redeployment training is mindfulness (sati) meditation, or mindful awareness training (Siegel, 2020). Sati meditation is all about paying attention to one’s own perception, sensations, feelings in and out of the body, that happen in the here and now. In sati meditation, one is supposed to pay clear attention not only to the sensations themselves, but also to the ways in which the sensations are processed by the self. With my eyes, I am looking at the current events unfolding in front of me, and at the same time, I am also looking, with my mind, at the fact that I am experiencing the events. In short, it is about experiencing those experiences.

For sati meditation, breathing is one of the most effective tools for attention training, because breathing is a continuous and never-ending event that is always happening to me, here and now, with or without my intention. Paying attention to breathing means paying attention to the bodily sensations that breathing brings about; namely, the subtle movements of the abdomen and breast, which often leads to relaxations of the muscles. Our intended attention to breathing, thus, leads us to pay attention naturally to the unusual interoceptive and proprioceptive sensations that one seldom experiences in everyday life (Cayoun and Shires, 2020). This is why sati meditation is an effective way of redeploying attention and why it has helped so many people with pains and negative emotions throughout history.

Another type of effective practice in sati meditation is body scan, which is paying attention to one’s own bodily sensations in real time. Researchers have confirmed that paying attention to one’s own bodily sensations, or “body awareness,” is a bottom-up process anchored in an interoceptive-insular pathway (IIP), which is intimately connected with autonomic and emotional brain areas, as well as verbal and non-verbal memory (Calsius et al., 2016). A proper manual body work may also activate this IIP and restore fascia covering the muscles and the internal organs, which are potent interoceptive generators.

Besides the redeployment of attention, implanting a new prediction model could be another effective way of treating chronic pain and emotional regulation disorders. If patients firmly believe that certain behaviors would relieve the pain, then they will get better from just enacting those behaviors; if you take a drug believing that it will work, or perform a “ritual of healing” that you believe it will work, active inference interprets even a slightest change in your interoceptive sensations as a sign of “pain relief” and the pain will subside (Von Mohr and Fotopoulou, 2018). As a narrative paradigm, the patient’s “belief” is the generative model at the highest level of hierarchical inference. As a conscious inference, it contextualizes the processing at the lower levels—through attentional redeployment—as we see in placebo effects and “rituals of healing.”

To liberate the patients from emotional regulation disorder or chronic pain, it is necessary to change the automatic inference and story-telling processes and provide the patients with accurate and healthy guidelines for reinterpretation of their interoceptive data. In other words, it is necessary to bring about a change in deep inference that automatically interprets certain sensory information in a certain way and makes certain stories (such as pain, anxiety, or trauma) less plausible than alternative states of being. This rests on changing interpretations and regulatory predictions in the inference system. To achieve this, it is necessary to neutralize, or weaken, the pre-existing storyteller and introduce a new narrative. The question then is how to effectively install a new generative model. To answer that question, we need to consider the nature of inference as well as the structure of the inference process more closely.

## What Is “Inference?”

### Abduction: The Basic Logical Structure of Inference

Active inference tells us that our brain is actively and constantly producing perceptions, meanings, and stories about the world. The logical structure of this meaning-production process is what Peirce calls “hypothetical inference” or “abduction.” One of the purposes of the present paper is to formalize the logical structure of active inference as abduction.

In studying Aristotle’s logical argument types, Peirce (1994) discovered a less known type of argument, abduction, distinct from the well-known types—deduction and induction. The logical structure of deduction proceeds in the order of Rule (major premise) → Case (minor premise) → Result (conclusion). A familiar example is: If there is a rule that “All men are mortal,” and there is a case that “Enoch is a man,” then the conclusion “Enoch will die” is drawn as a result for sure (Peirce, 1994, CP 2.620).

Abduction also starts with the Rule like deduction, “All men are mortal,” but it sees the Result, “Enoch is dead,” first and then it jumps to the conclusion, the Case of “Enoch is a man.” As we can see here, the conclusion of abduction is not quite as reliable as that of deduction or induction: Enoch is not necessarily a man; it could be the name of a dog or a cat. In fact, the nature of abduction is always a “fair guess,” a prediction, a probability, a hypothesis, or an inference.

Peirce believed that abduction was the same form of argument that Aristotle described “incompletely under the name of *apagögé* in Volume 2, Chapter 25 of Prior Analytics” (Peirce, 1994, CP 2.776). In fact, Aristotle’s argument named *apagögé* had almost disappeared from human history. According to Peirce, this was entirely due to “the stupid Apellicon” (Peirce, 1994, CP 5.144). After Aristotle’s death, his manuscripts did not see the light of the world for over 200 years. It was Apellicon of Teos, the rich book-collector, who bought the manuscripts for a large sum of money. Apellicon served as the first and self-appointed editor of Aristotle’s vast writings, supplementing damaged texts on his own.

According to Peirce, this “stupid Apellicon” put the wrong words in place of the unrecognizable words at his disposal; as a result, he made it impossible to understand what Aristotle’s *apagögé* was all about. Later, Aristotle’s writings were moved to Rome and passed on to Tyrannion, a renowned scholar and outstanding grammarian, who declared that “Apellicon’s editing was excessively bad” (Peirce, 1994, CP 7.234). Peirce argued that Aristotle evidently described the hypothetical inference under the term of *apagögé*, which had been dormant for more than 2,000 years. Peirce translated the term into an English word “abduction.”

### The Rule in Abduction as Prior Knowledge

Many good examples of abduction can be found in detective stories. By just glancing at a woman for the first time, Sherlock Holmes instantly knew that she was a typist (Doyle, 1892). Later, Holmes explained to his amazed friend Watson that he noticed two lines of traces in the woman’s sleeves, which meant that she would have pressed her wrists on the desk probably for typewriting. He told Watson that he relied as always on deduction; but actually, all of his reasoning was abductive, not deductive. He also emphasized that his reasoning relied on his abilities of observation in detail. But more precisely speaking, the core of Holmes’s wonderful reasoning lies in his ability to infer or interpret the meanings of the clues, or active inference, rather than in “observation.” Consider the structure of Holmes’s reasoning:

Rule – A lot of typewriting leaves two lines of traces on the sleeves.

Result – The woman’s sleeve shows two lines of traces.

∴ Case – The woman is a typist.

The Rule is the previous knowledge that Holmes already had (i.e., the prior beliefs of the generative model); Result is what he observed (i.e., incoming sensory data); Case is his conclusion based on his guessing, or inference (i.e., perception). This is a typical abduction. If Holmes did deduction, the orders of his reasoning would be Rule → Case → Result: in other words, Holmes already knew that the woman was a typist and concluded that her sleeves would have two lines of traces. If Holmes knows who the criminals are and reasons about the clues, then he does deduction. But Holmes always starts with the clues, interprets the meanings, and identifies the criminals, just like the brain’s active inference system. That is abduction.

In terms of certainty, deduction is secure and reliable, but produces no new information. On the other hand, abduction is weak, unreliable, and uncertain, and as such, it may produce new information. But it may go wrong. The woman was not a typist, and she had just borrowed the jacket from his sister; or she might have had some compulsive habit of pressing her wrists on the tables. In a sense, the real “adventure” that Holmes was willing to take was abduction.

According to Peirce, all scientific discoveries were also the results of abduction. For example, Kepler’s discovery of the planetary motions in elliptical orbits was also the result of abduction. The elliptical orbit was just one possibility out of many, if not infinite, possible shapes that could connect the observed positions of the planets. Many other shapes could have connected the points just like there were many possible other reasons why the sleeves had two lines of traces. Surprisingly, however, Kepler inferred, like Holmes, that the planets would move following the smooth and beautiful elliptical orbits. Kepler’s discovery was a product of adventurous and imaginative guessing, or abduction, rather than an inevitable conclusion of observation.

To process the abductive inferences, we need the Rule, or prior knowledge in Bayesian inference that corresponds to a prior probability distribution or belief. For example, to interpret the meaning of the wet road, we must have the knowledge, or the “Rule,” that “When it rains, the ground gets wet.” Only after that, when we come across the “Result” of “The ground is wet,” we may infer the “Case” that “It must have rained.” In other words, we interpret the meanings based on what we believe *a priori*. The same is true with the Markov blanket system’s general perception processes: the meanings and stories it produces from the sensory data all depend on its prior knowledge, or the Rule. If we want to change the ways in which the Markov blanket infers the meanings of sensory experiences, we need to change the “Rule,” or the internal models that generate predictions; especially, predictions of precision that underwrite sensory attenuation and attention.

### To Change the Inference Process, Change the Rule

The idea that our brain uses inference to perceive an object was first put forward some 150 years ago by physicist and physiologist Helmholtz, a little ahead of Peirce. Helmholtz conceptualized the unconscious and the automatic inference processes in visual perception as “unconscious inference” (Helmholtz, 1925/1867). According to Helmholtz, visual perception has its own rules beyond the control of human consciousness. To our eyes, the Sun appears to rise in the east and set in the west. Even if we know for sure that the Earth rotates and the Sun remains still, our knowledge does not affect our visual perception. The Sun always rises and sets. Helmholtz contends that the automatic and unconscious inference in perception evidently shows that sensory information is not assimilated by consciousness processes in our mind, but rather in the lower levels of the sensory nervous system.

Helmholtz argued that the unconscious inference was based on “induction.” Peirce also acknowledged the existence of the inference process in perception, but he challenged Helmholtz’s view by emphasizing that the logical structure of perception is not induction, but abduction (Peirce, 1994, CP 8.62–90). According to Peirce, to perceive a rose as a rose, the brain needs to do abduction. First, before seeing a rose, we need to already know that “roses have such and such shapes and colors” (Rule). Then, when we come across “something that looks like such and such” (Result), we apply our prior knowledge to the visual perception data and infer that “Oh, here is a rose” (Case). The prior knowledge, or the Rule as a prior probability distribution, is given by our accumulated previous experiences with roses and other flowers (Peirce, 1994, CP 3.642).

Helmholtz’s idea, which grasped the essence of the brain as an “inference machine,” was revived as one of the basic algorithms for machine learning through an article entitled “The Helmholtz machine” (Dayan et al., 1995). The idea that a generative model can affect sensory processing was first proposed by Helmholtz, so this model was called “Helmholtz machine” (Hinton et al., 1995). The implication of the Helmholtz machine is that if we can change the generative model, we may change the ways in which we interpret sensory data. Thus, we may treat chronic pain and emotional disorders by changing the generative models used by patients. In terms of Peirce’s abduction, changing the generative model means changing the “Rule” to be applied to the “Result” for a new “Case,” where the Rule is prior knowledge or belief, the Result is incoming interoceptive sensations, and the Case is the meaning of the sensory data.

To treat chronic pain and emotional disorders, the therapist should provide the patients with two different kinds of information: one is the sensory data to be processed unconsciously, and the other is a new generative model to be used in the top-down prediction process. The former can be given by hands-on, or manual touch, and the latter can be installed by verbal communication, to instantiate a shared narrative about the meaning of touch. In terms of abduction, the therapist would provide the Rule with conceptual form through verbal communication and the Result with manual touch at the same time. The therapist should provide novel sensory data that the patients are not familiar with, so that the patients’ generative model has to learn new explanations and new ways of inferring the causes of interoceptive stimuli (e.g., affiliative touch). This is why we need verbally guided touch in therapy. Now the question should be: Why guided touch is an effective way of altering the generative models, or installing the new “Rules,” in a patient’s consciousness?

## Guided Touch – an Effective Way of Intervening the Inference System

### Why Guided Touch (Touch With Verbal Communication)?

We would propose that the new generative models can be directly transferred from the internal states of the agent-practitioner to the internal states of the agent-patient *via* verbal communication without passing through the up-and-downs of hierarchical layers of the sensory-and-active states. Guided touch, or touch with verbal communication, is therapists’ verbal interactions with patients. It may take a form of simple explanation or casual conversation, but the essence of verbal guidance is generally to suggest new ways of inferring and interpreting.

Guided touch provides the patients with some sensory data along with the contextual information for interpretation in a healthier way. We may regard the verbal communication in guided touch as a dyadic exchange of linguistic communication, or “pure communication,” which would produce “a synchronization or alignment of belief states that circumvent any reference to external states of the world” (Friston et al., 2020).

In non-guided touch therapies, patients may fail to interpret the meaning of the sensory data as intended by the therapist. If, however, the therapist provides the patient with a guidance of verbal communication, then the patient may adopt the therapist’s verbal communication as a scaffold for a new generative model of how somatosensory and interoceptive sensations are generated—and attended to.

In touch with verbal guidance therapy, the therapist is supposed to simultaneously provide the sensory signals to be processed through the bottom-up route (touch) *and* the top-down priors to be evinced through the top-down route (verbal communication). This could make a particularly effective touch therapy, as the purpose of touch is to help the patients to learn new habits of sense-making, under their Markov blankets. In terms of abduction, guidance with verbal communication is the “Rule” and touch is the “Result.” By providing the Rule and the Result simultaneously, guided touch would train the patient’s active inference system to establish a healthier explanation for – and assimilation of – interoceptive and proprioceptive sensory signals.

By replacement of generative models, we mean establishing a new set of (habitual) interpretations or hypotheses. In other words, equipping the patient with a new set of explanations for their sensations that goes beyond simply nuancing or “updating prior” beliefs under dysfunctional interpretations. We would argue that these new interpretations or hypotheses correspond to the “Rules” that underlie the generation of sensory input—rules that have to be abductively discovered. This is why a patient needs a therapist who enables the discovery of a new generative model. For the replacement of the generative models with new ones, we can consider one or a combination of the three strategies: the first is flattening prior beliefs; the second is altering the SoA; and the third is implanting a new set of “Rules” (prior knowledge for storytelling) for inference.

### Flattening Prior Beliefs

Guided touch consists of the two components: touch and talk. Thus, guided touch would work in both directions simultaneously: By providing the novel sensory data, touch intervenes in the prediction processes from lower to higher levels. By providing evidence for novel generative models, talk intervenes in the prediction processes from higher to lower levels; thereby applying selective pressure from bottom-up and top-down at the same time. In this situation, sensory signals caused by touch will be instantly, and possibly self-evidently, interpreted with the generative model installed under the therapist’s guidance. We can read this as “flattening prior beliefs,” following the concept of “self-flattening” (Limanowski and Friston, 2020). Guided touch would reduce the depth of active inference and “flatten,” and as such, attenuate the old habit of inference, or the generative model of the agency-patient. The flattening in question here corresponds to reducing the precision of prior beliefs such that all high-level explanations for narratives are afforded the same confidence; leading to a flat landscape of priors, in which one can more easily explore alternative hypotheses.

Regarding flattening prior beliefs, guided touch has similar effects to mindful meditation insofar as in both cases the precision of prior beliefs is reduced; thereby flattening the free energy landscape and allowing other priors to be explored (“flat priors” is a term from statistics which means, *a priori*, any hypothesis is equally likely). “Mindfulness” is a somewhat misleading translation of “*sati*,” which should be translated to “awareness.” Mindful meditation, or *sati*, refers to a structured set of training for paying attention to sensory signals, without invoking any mental commentaries, value judgment, or narrative. Redirecting attention or precision to sensory information implicitly reduces the precision of prior beliefs and therefore instantiates flat priors. In guided touch therapy, sensations supplied by the therapists’ touch attract attention and reduce the precision of pre-existing priors; thereby allowing alternative hypotheses to be explored. In other words, the patients would be led to pay bare attention, as in mindful meditation.

In sati practices, meditators attempt to observe how they feel at the present moment, physically and mentally, whatever it is as it is, without adding any interpretations, mental commentary, or narratives (Kabat-Zinn et al., 1985). The traditional *sati* meditation is all about “flattening” deep active inference processes, which attenuate the precision of narratives at the highest level, ego or self-consciousness—the allegedly fundamental source of human suffering. *Sati* practices also emphasize concentration of attention on “breathing with the whole body,” which is a very effective way of redeploying attention from deep to sensory levels.

Training for flattening or attenuating the precision of prior beliefs can be done with two types of sensory data: One is with on interoceptive sensations, by paying attention to the sensations generated by the heart, gut, or any other internal organs. Traditional *sati* meditation with breathing exercises usually aims at paying bare attention to these interoceptive sensations. The other is with proprioceptive sensations by paying attention to the feelings from one’s own limb or torso movements. Traditional Asian contemplative practices, such as tai chi, qigong, and yoga, all aim at paying bare attention to the proprioceptive sensations. Payne and Crane-Godreau (2013) created a program named Meditative Movement (MM) by combining qigong, tai chi, and yoga to show that this type of exercise is an effective treatment for depression and anxiety. Levine also created a trauma therapy program named SE based on the modern somatic exercises to show that paying attention to interoceptive and proprioceptive sensations is an effective therapeutic tool for trauma and chronic stress (Payne et al., 2015).

### Altering the Sense of Agency

The goal of therapeutic touch is to provide the patient with a new habit of predicting interoceptive and proprioceptive sensations. For that purpose, it is necessary to attenuate not only certain precisions but also functions of the agent-patient, or the storyteller of the patient. In other words, for an effective touch therapy, we want not only redeployment of attention but alteration of the patient’s generative models at the higher levels.

Self awareness is part of our generative models that generates a mental commentary on all that we experience—and creates intentions for all actions. To replace an existing generative model with a new one, we need to either weaken or flatten the existing generative model. In other words, it is necessary to suspend the existing routine of storytelling at least for a short period of time. The classical example of suspending the existing generative model can be found in hypnotic suggestions. The mechanism of hypnosis can be explained by active inference theory (Jensen et al., 2017; Jamieson, 2018). In hypnotic states, deep active inference is temporarily stopped; the self-consciousness, or the mental commentary producer, is also temporarily absent. The existing generative model has been discarded, but a new generative model is not yet ready. This is the best window of opportunity through which new storytelling can be installed. At this moment, if the hypnotist provides a suggestion, the internal model of the subject would adopt the suggestion as one’s own storytelling.

Being hypnotized means being in a state of accepting suggestions (stories) from outside as one’s own narrative. If a person’s existing generative model gets weakened or flattened, it is more likely the person is ready to entertain stories from outside—the person becomes more “suggestible” – and this is the moment of “the alteration of the sense of agency (SoA)” (Martin and Pacherie, 2019). If we can weaken the patient’s generative model, the guidance, or verbal communication, during touch would have stronger effects in replacing the old habit of interpretation. To install a new generative model, we need to weaken the existing generative models first. Then, the question is: How can we suspend, attenuate, or flatten the existing generative model?

It may sound surprising, but the answer lies in a “surprise.” We may consider guided touch within a therapeutic alliance as a form of dyadic interaction, like a conversation, which consists of exchanging verbal and non-verbal behaviors. The two parties in a dyadic interaction rely on the assumptions that the other party would cooperate following the conventions defined by their own relationships, common sense, and culture (Kim and Kim, 2008). A good example is the cooperative principle (Grice, 1975) that can be found in conversational communication in common social settings. In such a dyadic interaction, it is well-known that a sudden out-of-context behavior of the one party would cause a temporary suspension of the generative model of the other. Regarding this point, we may get an insightful clue from a technique of hypnosis and the tradition of Zen meditation.

#### Instant Hypnosis: A Way of Suspending Generative Models

Consider an instant hypnotic induction involving a handshake. When I meet someone, if I reach for a handshake, I know the person will respond by holding my hand gently and look into my eyes with a friendly smile. Assume that I am meeting you for the first time. When I greet you, if you come to me with a smile and start to extend your hand, I will instantly interpret that gesture as a sign of the beginning of the handshake; surely, I will also reach out my hand as an almost automatic response. At that moment, I am quite confident what will happen next: I predict a handshake.

The handshake process starts; when your hand and my hand are about to contact, my sensory states and active states—as well as my generative model at the various hierarchical levels—are all engaged in the expected handshake process with full of confidence in the predictions—soon my hand will get the sensations of the handshake, my arms will be shaken by the handshake, my eyes will see your smile of handshake, my ears will hear your greeting words of handshake, and I will do all the necessary actions for the handshake. All my Markov blanket systems will engage in the familiar handshake process; all levels of the active inference systems are so confident about their predictions that they prepare little for any possible prediction errors.

At that very moment, you (hypnotist) suddenly grab my wrist, instead of my hand, gently but swiftly, and lift my hand to my face in a way that my palm faces my eyes in close proximity. Suddenly, I see nothing but my palm. If this happens very quickly, it will instantly produce massive prediction errors and overwhelming surprises beyond the control of my inference system; unexpected out-of-context sensory data are flooding in; I know the existing generative models are not working here anymore, so I must discard them; but what other generative models would be relevant here? How do I interpret this situation? I do not know. My whole active inference systems halt for a moment. This is the moment practically no generative models are functioning and deep active inference collapses. My prior beliefs become almost empty or flat—nothing but the discarded generative models, weakened agency, and muted ongoing storyteller (self-consciousness).

This is the very moment when I come extremely suggestible—my generative models can be easily replaced by the other party’s suggestions. So, hypnotists have been using this sort of window for the instant induction for a long time. Probably hypnotists do not have the concepts, nor the intention, of flattened generative models or emptied agency. But our active inference model can explain quite reasonably why the “confusion techniques” like “handshake instant induction” have been effective for hypnosis (Erickson, 1964).

#### Zen: Another Way of Suspending the Generative Model

Interestingly, the abrupt out-of-context linguistic interactions are at the core of traditional Zen meditation, the ultimate goal of which is to achieve enlightenment through discarding the ego. In Koan, the legendary scriptures of the dialogues between the Zen masters and the monks, most conversations conclude with the masters’ abrupt and seemingly out-of-context answers and behaviors. Some of the well-known examples are:

A monk asked Zhaozhou, the famous Zen master:

“What is the meaning of Bodhidharma’s coming to China from the West (India)?”

Zhaozhou answered: “There is a cypress tree standing in the front garden” (Heine, 2020).

Another example:

A monk asked Master Dongshan: “What is Buddha?”

And the master answered: “Three pounds of flax” (Kubose, 1973).

It is said that the monks were suddenly enlightened after soliciting these nonsensical answers. Besides these kinds of dialogues, the masters would suddenly yell at the monks, slam the door in the face and walk away, or even unexpectedly beat the monks with a stick. After these nonsensical out-of-context interactions, the monks would instantly be enlightened. In the Zen tradition, that is the moment of enlightenment, when the pre-existing ego (the storyteller, self-consciousness, or in our terms, the pre-existing generative model) instantly vanishes, and the monks realize the true self with an altered SoA.

The point here is not to suggest that hypnosis or Zen meditation should be incorporated into guided touch therapy. The goal of guided touch is in no way like hypnosis nor to Zen. We are just exploring the possibilities of guided touch in weakening the patient’s generative models and altering her SoA. The instant hypnotic induction technique with handshake and the traditional Zen stories suggests that touch with some out-of-context verbal communication may effectively attenuate the patient’s agency and facilitate the replacement of the patient’s pre-existing generative models.

### Implanting a New Set of “Rules” for Inference: Placebo as Inner Communication

As we have seen in the structure of abduction, inference starts with the “Rule,” or the prior knowledge for interpretation and storytelling. The “Rule” usually takes the form of inner communication. Something taken for granted is a good example of the “Rule” in abduction. The studies on placebo and nocebo effects have shown that inner communication can influence the ways in which the human body reacts to stimuli such as new drugs and treatments (Mommaerts and Devroey, 2012).

Inner communication has both characteristics of stories and thoughts. Thinking to oneself–or, “verbal thought” – is an archetype of inner communication along with talking to oneself, in the sense of an internal narrative (Vygotsky, 2012). Inner communication may take various forms–such as thoughts, beliefs, mental commentaries, inner voice, intrapersonal communication, and imagined interactions (Honeycutt, 2002). More often than not, inner communication is not something that we do intentionally; rather, it is the inner narrative we entertain, with or without our intentions; our thoughts are not what we do, but rather what happens to us. As such, we cannot possibly plan exactly what we will be thinking in five minutes from now. The same is true with what we believe. These “beliefs” or “thinking to oneself” are the fundamental basis of placebo and nocebo effects.

Many studies have shown that placebos change the body, not just psychologically, but physiologically and biologically. The biggest challenge in developing a new drug is to beat placebos, which almost always have strong and significant effects. It is no joke to say that placebos are the most effective, safe, and side-effect free drugs.

Placebos can reduce pain to the same extent as morphine. In a double-blind placebo study with 74 patients—who underwent extraction of impacted mandibular third molars—placebo (saline), 4, 6, 8, 12 mg of morphine were administered. The degree of pain relief by the placebo was found to be equivalent between 4 and 6 mg of morphine (Levine et al., 1981).

Placebos have proven their effectiveness in surgery as well as in medicine. In a double-blind study with a total of 180 patients—with osteoarthritis of the knee—were randomly divided into the three groups to receive arthroscopic débridement, arthroscopic lavage, and placebo surgery, respectively. Patients in the placebo group received skin incisions and underwent a simulated débridement without insertion of the arthroscope. As a result of follow-up for 2 years, the mean scores on the Knee-Specific Pain Scale were similar across the three groups. Furthermore, all three groups showed similar knee health recovery, and walking and stair climbing were also performed to a similar degree (Moseley et al., 2002).

Placebo effects depend on what stories the participants tell themselves: inner communication matters. In a double-blind study with 82 healthy paid volunteers, a new pain reliever (purported opioid analgesic) was administered. All participants received identical placebo pills, but half of the participants were informed that the drug had a regular price of $2.50 per pill and the other half that the price had been discounted to $0.10 per pill. Electrical shocks to the wrist were given twice for each participant, before and after taking the pill, to assess the change in pain. Reduction in pain was reported by 85.4% of the regular-price group and by 61.0% of the discounted price group (Waber et al., 2008). The result shows that simple beliefs, or inner communication, that “expensive pills would have stronger effects” caused the difference.

The belief that the medications one takes may have harmful side effects also has a powerful effect. If placebo takers are warned of possible negative effects, say, “This is a newly developed medication for lowering blood pressure, but it might cause headaches and stomach pains for some people,” then many of them would report headaches and stomach pains. This is a nocebo effect (Hansen and Zech, 2019). If one is certain he is going to die soon, his longevity is shortened. If people of old age believe that they will soon get sick and die, they appear to have a shortened lifespan. According to the Ohio Longitudinal Study of Aging and Retirement (OLSAR), people with negative prejudices against aging shortened their life expectancy by an average of 7.5 years. Those who had positive self-perceptions of aging, like “Things are getting better (not worse) with aging”; “I am as good as last year,” and so on, lived longer and the difference was statistically significant (Levy et al., 2002).

The phenomenon that one’s inner communication has a powerful effect on one’s own body suggests that the stories and the meanings produced by the consciousness, or the generative model at the highest level, would influence the inference processes at the lower levels. This means that the patient’s thoughts about the effect of touch therapy will have a significant bearing on the outcome of touch therapy. Here, the patient’s thoughts are a “Rule” of the abduction that determines how the patient interprets the sensory information provided by touch therapy. Once the existing generative model is weakened or suspended, a new generative model, or new Rules for abduction can be installed, and the content of the Rule should reflect the desired effects of the touch therapy.

## Hypotheses and Suggested Treatment Conditions for Experimental Studies

### Treatment Conditions for Experimental Studies

For investigating the effects of the guided touch through experimental studies, we propose the following treatment conditions:

#### Treatment 1: Control

The therapist offers simple verbal descriptions regarding the touch. This type of treatment would work as the control condition.

For example: “Now we are releasing the tension of this group of muscles”

“This touch will relax your right trapezius.”

#### Treatment 2: Redeployment of Attention

To realign the precision or gain control mechanism of active inference, the therapist asks a series of questions directing the patient’s attention to a wide range of focal points. The patients will be told that they do not have to answer the questions verbally. The patients will be encouraged to pay clear and distinct attention, or bare attention, to the mentioned parts of their bodies.

For example: “How do you feel here?”

“Can you recognize the difference between this and that?”

#### Treatment 3: Flattening Prior Beliefs

In this treatment, the sensory signals caused by touch will be instantly interpreted under the generative model installed *via* the therapist’s verbal guidance. The therapist will provide verbal guidance and touch simultaneously; the patient will update their interpretational framework in the form of imageries, which would allow the patient to interpret the touch signals in a novel way.

For example: “Imagine this part of fascia getting relaxed and elongated like an old rubber band.”

“Your lower back is now getting full of soft and colorful bubbles that are gently floating around.”

#### Treatment 4: Weakening Pre-existing Generative Models

Sudden and abrupt out of context comments will induce irresolvable prediction errors, and as a result, attenuate the precision of prior beliefs. Right before the “out of context comments,” the therapist will give some usual and familiar explanations. The underlined sentences are the “out of context comments.” Actual touch will temporarily stop while out of context verbal communication is provided; however, immediately after the out of context comments, when the patient becomes more suggestible, a new set of interpretational frameworks will be given along with touch.

For Example: “Today we will focus on your lower back muscles…. How do you feel these days?

They say there is a calm desert on the dark side of the moon.”

“Sure, your lower back muscles have very strong tension. I once had a white Labrador, but now I have a golden retriever.”

#### Treatment 5: Installing New Generative Models

At the highest level of the active inference system, or self-consciousness, the generative model exists in the form of ongoing inner communication. An effective way of replacing the pre-existing generative models with a new narrative would be guided inner communication. With their manual touch, the therapists would ask the patients to silently repeat—three times—what they say sentence by sentence. The patients are supposed to do the inner communication within their mind only without necessarily speaking out, but whispering would be also fine.

For Example: “Repeat after me silently, only in your mind”

“I am happy now” “I am relaxed.”

“I am feeling the warm sensations on my shoulder.”

“I am looking at the source of the pain in my shoulder.”

“I see the pain is fading away.”

#### Dependent Variables

The possible effects to be measured, or the dependent variables are as follows: self-report measures on psychological and behavioral variables (affective scales, behavioral responses, etc.) and subjective relief from chronic pain (Von Korff et al., 1992); biomarkers related to the autonomic nerve system and emotional responses such as heart rate, heart rate variability indices (Appelhans and Luecken, 2006), galvanic skin response, and EMG; and responses to artificially induced pain (Yucel et al., 2005) or inflammation (Rosenkranz et al., 2013).

### Hypotheses for Experimental Studies

One can design multiple experimental studies by combining the treatment conditions above. Based on active inference and the prediction model, the present paper proposes the following hypotheses:

**H1:** Guided touch therapies inducing redeployment of attention (Treatment 2) would show stronger effects in relieving chronic pain and negative emotions than touch with simple verbal descriptions (Treatment 1).

**H2:** Guided touch therapies which “flatten” prior beliefs (Treatment 3) would show stronger effects in relieving chronic pain and negative emotions than touch with simple verbal descriptions (Treatment 1).

**H3:** Our assumption is that the “out of context comments” would make patients more suggestible by attenuating the precision of pre-existing generative models (i.e., prior beliefs), and consequently, would increase the effect size of Treatment 5. Thus, the effect size of Treatment 5 immediately after Treatment 4 would be greater than that of Treatment 5 immediately after Treatment 1.

## Conclusion

According to active inference theory, the essence of touch in manual therapy is the provision of sensory data for (re)interpretation by a patient. To teach the patient a new way of interpreting interoceptive and proprioceptive signals, therapists should install a complementary set of prior beliefs—to revise generative models—through verbal communication.

The nature of inference is abduction. To change the conclusion of an inference, it is necessary to change the “Rule,” or the prior knowledge underwriting the generative model: pre-existing generative models should be replaced with new narratives. To facilitate such replacement, the present paper proposes that we should weaken pre-existing generative models, through flattening prior beliefs, altering the SoA, and enabling a redeployment of attention. Then, a new generative model can efficiently be installed through inner communication, along with touch as the sensory evidence for this new narrative.

Based on these arguments, we propose several hypotheses for a series of empirical studies, which would test not only our theories but the relevance of active inference in general at the behavioral level. If the behavioral studies support our hypotheses, one could pursue further studies, investigating functional connectivity among distributed neuronal responses (Kyeong et al., 2017, 2020).

Active inference and predictive coding models are currently shaping behavioral sciences as well as neuroscience (Hutchinson and Barrett, 2019). Recent studies on meditation, for example, have already incorporated the active inference perspectives (Lutz et al., 2019; Deane et al., 2020; Sandved-Smith et al., 2021). If touch with verbal guidance is proven to be effective, that would facilitate new approaches based on the active inference model for studying not only meditation but hypnosis, placebo, and self-consciousness as a storyteller. Furthermore, it would open up new possibilities of employing inner communication interventions, including self-talk training, for a wide range of mental as well as physical therapies (Kim et al., 2021).

Some of the other significant implications are as follows: First, it opens new theoretical horizons for traditional therapies and psychiatric treatments in general. A deep active inference perspective suggests that therapies and psychiatric treatments, whether talk-based, touch-based, cognitive or behavioral, all fundamentally depend on how patients’ bodies and minds interpret therapists’ messages, whether they are given through verbal communication or touch sensations. The concepts of attenuation of gain control, revision or replacement of generative models, flattening prior beliefs, weakening the generative models and altering the SoA would have significant implications for various types of therapies treating PTSD, anxiety disorder and depression, especially involving somatic movements and interoceptive meditation.

Second, as the guided touch aims at establishing “new” ways of sense-making and “new” corporeal narratives, this study would also have some implications for dopaminergic circuit related studies, ranging from reinforcement theory and motivation to Parkinson’s and Alzheimer’s disease. Friston et al. (2009) once suggested that “dopamine (encoding) does not encode the prediction error of value but the value of prediction error”. And “dopamine balances bottom-up sensory information and top-down prior beliefs when making hierarchical inferences (predictions) about cues that have affordance…. [W]e can confuse agents by changing the context (order) in which cues are presented” (Friston et al., 2012). Some of the recent studies support this view: FitzGerald et al. (2015); Gardner et al. (2018), Gershman and Uchida (2019). As such, the sudden “out of context” interactions should have some relevance to the dopamine system and its encoding of the “value of prediction error,” as they are novel stimuli provoking massive prediction errors.

Third, by combining imagery, inner communication, and “out-of-context” (intended surprise) methods, this study may open new possibilities for affective guided-based touch techniques for proprioceptive and interoceptive multi-modal stimuli. And these new touch techniques would have some theoretical as well as practical implications for the deep active inference in shared and synchronized Markov blankets.

Finally, if this study demonstrates some effectiveness of guided touch, we may produce a series of standardized guided touch programs with recorded voices of experienced and renowned therapists. If therapists use the recorded guidance, while they perform their manual touch cares, patients will receive quite standardized high quality guided touch therapy. In the long run, we hope, the standardization of the guidance will lead the field toward the standardization of manual touch practices as well. This could make a milestone for developing standardized frameworks for therapeutic alliance in the fields of chronic pain and emotional disorder.

## Data Availability Statement

The original contributions presented in the study are included in the article/supplementary material, further inquiries can be directed to the corresponding author.

## Author Contributions

JK wrote the first draft of the manuscript, which KF thoroughly revised and edited. All authors contributed to the conception of the manuscript, first discussion from which the main idea of this manuscript emerged, revised the manuscript, and approved and were accountable for the submitted manuscript.

## Conflict of Interest

JE was employed by the Malta ICOM Educational Ltd. The remaining authors declare that the research was conducted in the absence of any commercial or financial relationships that could be construed as a potential conflict of interest.

## Publisher’s Note

All claims expressed in this article are solely those of the authors and do not necessarily represent those of their affiliated organizations, or those of the publisher, the editors and the reviewers. Any product that may be evaluated in this article, or claim that may be made by its manufacturer, is not guaranteed or endorsed by the publisher.
